# Re-treatment with etanercept is as effective as the initial firstline treatment in patients with juvenile idiopathic arthritis

**DOI:** 10.1186/s13075-021-02492-0

**Published:** 2021-04-16

**Authors:** Jens Klotsche, Ariane Klein, Martina Niewerth, Paula Hoff, Daniel Windschall, Ivan Foeldvari, Johannes-Peter Haas, Gerd Horneff, Kirsten Minden

**Affiliations:** 1 German Rheumatism Research Centre, Leibniz Institute, 10117 Berlin, Germany; 2 Centre for Paediatric Rheumatology, Department of Paediatrics, Asklepios Clinic Sankt Augustin, Sankt Augustin, Germany; 3 Department of Pediatrics, Medical Faculty, University of Cologne, Cologne, Germany; 4 Endokrinologikum Berlin, 10117 Berlin, Germany; 5 Department of Paediatric and Adolescent Rheumatology, North-Western German Centre for Rheumatology, St. Josef-Stift Sendenhorst, Sendenhorst, Germany; 6 Hamburg Centre for Pediatric and Adolescent Rheumatology, Hamburg, Germany; 7 German Center for Pediatric and Adolescent Rheumatology, Garmisch-Partenkirchen, Germany; 8 Department of Rheumatology and Clinical Immunology, Charité - Universitätsmedizin Berlin, Berlin, Germany

**Keywords:** Juvenile idiopathic arthritis, Etanercept, Effectiveness, Disease flare, Window of opportunity, Inactive disease, Remission

## Abstract

**Objectives:**

To determine (i) correlates for etanercept (ETA) discontinuation after achieving an inactive disease and for the subsequent risk of flare and (ii) to analyze the effectiveness of ETA in the re-treatment after a disease flare.

**Methods:**

Data from two ongoing prospective registries, BiKeR and JuMBO, were used for the analysis. Both registries provide individual trajectories of clinical data and outcomes from childhood to adulthood in juvenile idiopathic arthritis (JIA) patients treated with biologic disease-modifying anti-rheumatic drugs (bDMARDs) and conventional synthetic DMARDs (csDMARDs).

**Results:**

A total of 1724 patients were treated first with ETA treatment course (338 with second, 54 with third ETA course). Similar rates of discontinuation due to ineffectiveness and adverse events could be observed for the first (19.4%/6.2%), second (18.6%/5.9%), and third (14.8%/5.6%) ETA course. A total of 332 patients (+/−methotrexate, 19.3%) discontinued ETA after achieving remission with the first ETA course. Younger age (hazard ratio (HR) 1.08, *p* < 0.001), persistent oligoarthritis (HR 1.89, *p* = 0.004), and shorter duration between JIA onset and ETA start (HR 1.10, *p* < 0.001), as well as good response to therapy within the first 6 months of treatment (HR 1.11, *p* < 0.001) significantly correlated to discontinuation with inactive disease. Reoccurrence of active disease was reported for 77% of patients with mean time to flare of 12.1 months. We could not identify any factor correlating to flare risk. The majority of patients were re-treated with ETA (*n* = 117 of 161; 72.7%) after the flare. One in five patients (*n* = 23, 19.7%) discontinued ETA again after achieving an inactive disease and about 70% of the patients achieved an inactive disease 12 months after restarting ETA.

**Conclusion:**

The study confirms the effectiveness of ETA even for re-treatment of patients with JIA. Our data highlight the association of an early bDMARD treatment with a higher rate of inactive disease indicating a window of opportunity.

## Key messages

- An early bDMARD treatment was associated with a higher likelihood to achieve inactive disease state and discontinue etanercept, thus indicating a window of opportunity.
- The study confirms the high flare rate in juvenile idiopathic arthritis after discontinuing treatment. No sub-cohort could be defined without this risk or with a lower flare risk.
- Patients who were re-treated responded well to etanercept in the second treatment course after discontinuing etanercept by inactive disease.

## Introduction

Juvenile idiopathic arthritis (JIA) is the most common chronic inflammatory rheumatic disease in children and adolescents. According to the currently valid International League of Associations for Rheumatology (ILAR) classification [1, 2], JIA comprises six different forms of arthritis that begin before the age of 16 and differ from each other in clinical presentation, course, response to therapy, genetic background, and extra-articular manifestations. They all share one feature: consistent therapy is required to avoid consequential damage and permanent loss of function. Biologic disease-modifying anti-rheumatic drugs (bDMARDs) provide a well-accepted treatment option for patients with a severe course of JIA and those who do not respond or are intolerant to conventional synthetic DMARDs (csDMARD) such as methotrexate (MTX) [3, 4]. To date, several different bDMARDs targeting different cytokines are available for the rheumatologist to control disease activity, induce an inactive disease, or even cause remission in a patient with a severe course of JIA. The main treatment principles include tumor necrosis factor (TNF)-α receptor antagonists (etanercept (ETA), adalimumab (ADA), golimumab, the interleukin (IL)-6 pathway inhibitor (tocilizumab (TOC)), and IL-1 pathway inhibitors (anakinra and canakinumab)), and the T-cell co-stimulatory signal modulator abatacept, among others.

ETA is the most commonly prescribed bDMARD for the treatment of patients with JIA [5, 6]. To date, ETA has shown good treatment response in clinical trials [7, 8] and observational studies [9–11] as well as good tolerability [5, 7, 8, 11, 12] in patients with JIA. This information is mainly based on the first treatment course with ETA [7, 8, 10, 11], except for Otten et al. [13] and Horneff et al. [9], who analyzed the effectiveness of ETA in a second or third line treatment. Approximately 15% of children and adolescents could discontinue ETA after achieving an inactive disease [9, 14]. However, some JIA patients fail to respond to ETA and switch to another bDMARD [13, 15]. Scant knowledge exists about the disease course and treatment patterns after discontinuation of the first ETA treatment course. Otten et al. [13] were the first to report on the effectiveness and treatment patterns of the second and third bDMARDs after discontinuation of ETA in JIA patients. They concluded that switching of bDMARDs is common in JIA patients. However, the study only tracked a small number of patients in the second and third treatment courses.

The aims of the current study are to (i) evaluate therapy survival of treatment with ETA for the first, second and third treatment course, (ii) investigate the correlates of treatment discontinuation of ETA due to ineffectiveness and after achieving an inactive disease, (iii) investigate the rate of reoccurrence of active disease as well as its correlates after discontinuation of ETA due to an inactive disease, and (iv) assess the clinical course and response to treatment after restarting ETA. Data of the German JIA biologic register, Biologika in der Kinderrheumatologie/Biologics in Paediatric Rheumatology (BiKeR), and its follow-up register in adulthood, Juvenile arthritis Methotrexate/Biologics long-term Observation (JuMBO), were used for this analyses.

## Patients and methods

Data from the two ongoing prospective, multicenter, non-interventional cohort studies, BiKeR and JuMBO, were used for the analyses. The pediatric register BiKeR was launched in 2001 to monitor the safety and effectiveness of bDMARDs and csDMARDs in the routine rheumatologic care of children and adolescents with JIA. JuMBO is the follow-up study to BiKeR, and follows the BiKeR patients who left pediatric care or have reached the 18th year of life. Both registers provide individual trajectories of clinical data and outcome data from childhood to adulthood in patients treated with bDMARDs and csDMARDs.

### Patients

Children and adolescents with a definite diagnosis of JIA defined by the ILAR criteria [1, 2] and starting treatment with a bDMARD or MTX monotherapy during childhood were consecutively enrolled in the BiKeR registry. Our inclusion criterion covered all BiKeR patients over 18 years (*n* = 2584) to include the subset patients who were potentially available for enrolment in JuMBO. The observation period covered for each patient the total follow-up period from enrolment in BiKeR (childhood) until the last available follow-up in adolescents in JuMBO. Among those, we selected 1779 JIA patients who had received at least one ETA dose for our analysis. All patients who were never treated with ETA during the observation period in BiKeR and JuMBO were excluded from this study.

Written informed consent was obtained from both the parents and the patients (age ≥ 8 years) for participation in BiKeR, and again from the patients (age ≥ 18 years) for further follow-up in JuMBO. BiKeR was approved by the Ethics Committee of the Medical Council of North Rhine-Westphalia, Duesseldorf, Germany (approval number 6000201531). JuMBO was approved by the Ethics Committee of Charité University Medicine Berlin (approval number: EA1/084/07). Both registers are conducted in accordance with the Declaration of Helsinki.

### Assessment

At baseline, in BiKeR, the pediatric rheumatologist documented the date of JIA onset, JIA category, antinuclear antibody (ANA) status, and presence of the human leukocyte antigen (HLA). Thereafter, patients were prospectively assessed after 3 months and then 6 monthly with a standardized case report form completed by the physicians and patients in BiKeR and JuMBO. The physician reported the start and end dates as well as the reasons for discontinuation for each treatment course with csDMARDs/bDMARDs. Discontinuing ETA after reaching an inactive disease was based on the physician’s decision and the Wallace criteria [16]. All bDMARD and MTX treatment episodes were identified in both registers. The number of treatment episodes with ETA and all treatment episodes before each ETA treatment interval were calculated for each single ETA treatment course. For each BiKeR and JuMBO visit, the physicians reported the current disease activity (global assessment) on a visual analogue scale (VAS), a 72-joint count (including the number of swollen, painful and limited of motion (LOM) joints), levels of C-reactive protein (CRP), and the erythrocyte sedimentation rate. The patient-reported outcomes included the evaluation of overall well-being and pain levels on a 10-cm VAS in addition to completed childhood health assessment questionnaires (CHAQ) [17] by the parents or adolescents in BiKeR. The young adults in JuMBO independently reported the patient outcomes and their disabilities in a health assessment questionnaire [18]. If a patient had uveitis during the course of the disease, then the physician reported the potential uveitis flares (active uveitis) in the adverse event form. The clinical Juvenile Arthritis Disease Activity Score in 10 joints (cJADAS-10) [19] was additionally calculated to assess disease activity. In the analyses of patients’ disease trajectories, inactive disease was defined by either the best possible rating on physicians’ global assessment of disease activity (< 1) or a cJADAS-10 score lower than or equal to 1 [19].

### Statistical analyses

We reported the sociodemographic and clinical characteristics of patients treated with ETA as the first biologic in the JIA disease course (first ETA course) and for patients treated with the second and third ETA courses that did not correspond to the second or third bDMARD in the treatment history. Therapy discontinuation was examined using Kaplan–Meier methods separately for patients who discontinued ETA after achieving an inactive disease or for patients who discontinued ETA due to ineffectiveness. Cox-proportional hazard models were applied to analyze the potential correlates for ETA discontinuation due to inactive disease or ineffectiveness. Harrel’s *c*-statistics (range 0.5 to 1; higher values indicate better predictive performance) were calculated for each predictor variable in the univariate analysis as a measure of predictive performance. The likelihood to discontinue ETA due to inactive disease was additionally analyzed by a multivariable Cox-proportional hazard model based on the results of the univariate analyses. The risk of reoccurrence of active disease after ETA discontinuation due to inactive disease was analyzed by a Cox-proportional hazard model. The effectiveness of ETA in the first treatment course was examined by linear mixed models using the physicians’ global assessment of disease activity, cJADAS-10 score, the number of joints with active arthritis and CRP levels in the first 24 months after the treatment started. All patients initiating treatment with ETA were included in the analyses (following the intent-to-treat principle). The generalized linear mixed models were used to calculate the predicted means of cJADAS-10, number of joints with active arthritis and presence of CRP for all JIA patients, and separately for the eight JIA categories. The same approach was applied to show the effectiveness after the restart of ETA after its discontinuation due to inactive disease and subsequent reoccurrence of active disease requiring bDMARD therapy. A *p* value < 0.05 was considered statistically significant. Statistical analysis was conducted with SAS, version 9.4.

## Results

### Patients and disease characteristics

A total of 1779 patients (68.8% of 2584) were ever treated with ETA in our cohort, providing 2178 treatment courses. The second-most frequent number of patients was treated with ADA (*n* = 646) followed by TOC (*n* = 227). ETA was the first bDMARD used in 1724 patients at BiKeR enrolment. The mean follow-up was 6.3 (standard deviation (SD) 4.6) years for the total cohort (*n* = 2584) and 8.6 (SD 4.2) years for the subset of patients with at least follow-up visit JuMBO (adulthood, *n* = 1535). The sociodemographic and clinical characteristics at start of the first (*n* = 1724), second (*n* = 338), and third (*n* = 54) ETA treatment courses are reported in Table 1. In general, the mean disease activity and mean levels of patients reported outcomes were higher at start of the first ETA course as compared to those at the start of the second and third courses.

**Table 1:** Patient characteristics at start of treatment with etanercept

	1st ETA course, *n* = 1724	2nd ETA course, *n* = 338	3rd ETA course, *n* = 54
Female gender	1146 (66.5%)	224 (66.3%)	32 (59.3%)
Age, years, mean (SD)	13.4 (3.5)	15.9 (4.3)	17.1 (5.1)
< 10 years	231 (13.4%)	17 (5.0%)	1 (1.9%)
10 to < 15 years	732 (42.5%)	97 (28.7%)	16 (29.6%)
> 15 years	761 (44.1%)	224 (66.3%)	37 (68.5%)
JIA categories			
Systemic JIA	111 (6.4%)	22 (6.5%)	4 (7.4%)
Polyarticular arthritis, RF-negative	487 (28.3%)	100 (29.6%)	21 (38.9%)
Polyarticular arthritis RF-positive	176 (10.2%)	25 (7.4%)	4 (7.4%)
Persistent oligoarthritis	73 (4.2%)	14 (4.1%)	1 (1.9%)
Extended oligoarthritis	307 (17.8%)	69 (20.4%)	9 (16.7%)
Enthesitis-related arthritis	359 (20.8%)	65 (19.2%)	9 (16.7%)
Psoriatic arthritis	148 (8.6%)	33 (9.8%)	2 (3.7%)
Undifferentiated arthritis	63 (3.7%)	10 (3.0%)	4 (7.4%)
ANA positive	723 (43.9%)	134 (39.8%)	19 (35.2%)
HLA-B27 positive	472 (30.3%)	103 (30.6%)	14 (25.9%)
Physician’s global assessment, VAS score, mean (SD)	5.3 (2.7)	3.2 (2.8)	2.9 (2.5)
*CRP, mg/dl, mean (SD)*	19.5 (36.1)	12.5 (27.1)	10.5 (17.3)
*ESR, mm/1 h, mean (SD)*	24.6 (23.7)	19.2 (19.2)	15.8 (15.3)
Number of joints with arthritis, mean (SD)	7.2 (8.7)	3.7 (7.6)	2.8 (3.8)
Number of LOM joints, mean (SD)	8.0 (9.8)	4.9 (9.4)	3.5 (3.9)
Number of swollen joints, mean (SD)	5.7 (7.9)	2.6 (6.5)	2.0 (2.9)
Number of painful joints, mean (SD)	7.2 (9.1)	3.8 (7.8)	3.3 (4.4)
cJADAS-10, mean (SD)	15.1 (6.8)	8.4 (7.0)	7.8 (6.8)
CHAQ total score, mean (SD)	0.7 (0.6)	0.4 (0.6)	0.3 (0.4)
Patient-reported overall well-being, VAS score, mean (SD)	4.7 (2.8)	3.0 (2.7)	2.5 (2.6)
Patient-reported pain, VAS score, mean (SD)	4.2 (2.8)	3.1 (2.8)	2.7 (2.7)

*ANA* antinuclear antibodies, *bDMARD* biological disease-modifying anti-rheumatic drug, *CHAQ* Childhood Health Assessment Questionnaire, *cJADAS* clinical Juvenile Arthritis Disease Activity Score, *CRP* C-reactive protein, *ESR* erythrocyte sedimentation rate, *HLA* human leukocyte antigen, *LOM* limitation of motion, *RF* rheumatoid factor, *SD* standard deviation, *VAS* visual analogue scale

Two in three patients (*n* = 1197, 69.4%) were treated with ETA in combination with MTX when starting ETA in the first course, whereas 631 patients (36.6%) were treated with ETA in combination with MTX on the date of discontinuation of ETA or the date of the last follow-up, whichever came first (Table 2). MTX was discontinued (*n* = 566) after achieving an inactive disease (7%), onset of an AE (7%), other reasons (3%), ineffectiveness (1%), and unknown (82%). The rate of patients treated with a combination of ETA and MTX differed between the JIA categories (52.9% for enthesitis-related arthritis to 80.7% for RF-positive polyarthritis). The number of patients treated with ETA in combination with MTX was remarkably lower at the start of the second (133, 39.4%) and third (*n* = 15, 27.6%) treatment courses. In addition, the mean time under combination therapy decreased with each additional ETA course (1st, 1.5 years (SD 1.6), to 3rd, 0.2 years (SD 0.3)).

**Table 2:** Concomitant therapy with methotrexate at start of treatment with etanercept

	1st ETA course, *n* = 1724	2nd ETA course, *n* = 338	3rd ETA course, *n* = 54
All JIA	1197 (69.4%)	133 (39.4%)	15 (27.6%)
Systemic JIA	91 (82.0%)	12 (54.6%)	3 (75.0%)
Polyarticular arthritis, RF-negative	370 (76.0%)	40 (40.0%)	7 (33.3%)
Polyarticular arthritis RF-positive	142 (80.7%)	9 (36.0%)	2 (50.0%)
Persistent Oligoarthritis	50 (68.5%)	8 (57.1%)	0 (0.0%)
Extended Oligoarthritis	204 (66.5%)	25 (36.2%)	1 (11.1%)
Enthesitis-related arthritis	190 (52.9%)	25 (38.5%)	1 (11.1%)
Psoriatic arthritis	109 (73.7%)	10 (30.3%)	1 (50.0%)
Undifferentiated arthritis	41 (65.1%)	4 (40.0%)	0 (0.0%)
MTX at ETA stop/last observation	631 (36.6%)	79 (23.4%)	8 (14.8%)
Ever concomitant treatment with MTX	1239 (71.9%)	144 (42.6%)	15 (27.8%)
Duration of concomitant MTX therapy since start of ETA in patients who stopped MTX in years, mean (SD)	1.5 (1.6)	0.8 (1.5)	0.2 (0.3)

*ETA* etanercept, *MTX* methotrexate, *RF* rheumatoid factor, *SD* standard deviation

### Therapy survival of ETA

**Fig. 1:**
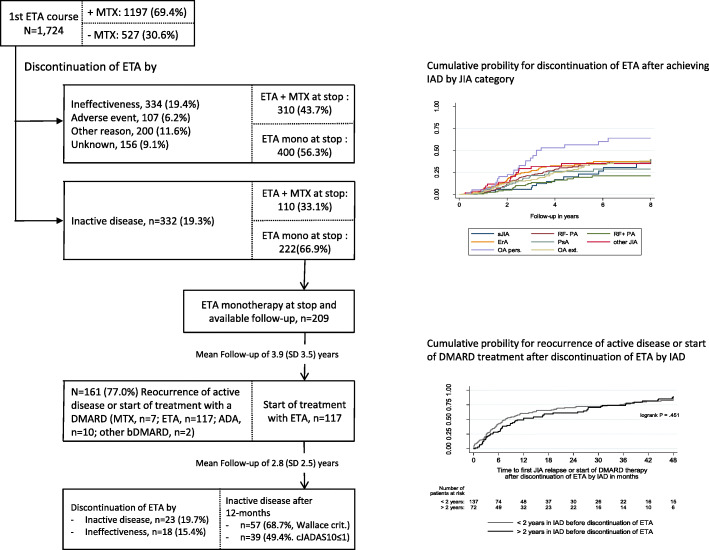
First etanercept treatment course, therapy survival and reasons for discontinuation and follow-up of patients who discontinued etanercept after achieving an inactive disease (single categories (ineffectiveness, adverse events, other reasons, unknown) do not add up to the rate of other reasons than inactive disease by the possibility of multiple responses)

**Fig. 2:**
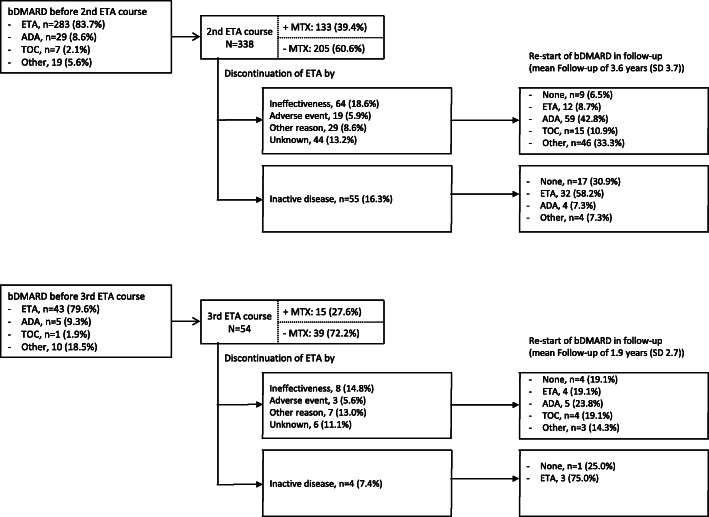
Second and third etanercept treatment course, therapy survival, and reasons for discontinuation (single categories (ineffectiveness, adverse events, other reasons, unknown) do not add up to the rate of other reasons than inactive disease by the possibility of multiple responses)

The mean treatment duration was 3.5 years (95% confidence interval (CI), 3.3; 3.7, maximum, 17.7 years) for the first treatment course, and 2.7 years (95% CI 2.4; 3.0) and 2.3 years (95% CI 1.7; 2.9) for the second and third courses, respectively. About 19.4% of the patients discontinued ETA due to ineffectiveness in the first treatment course, this proportion was comparable to the second (18.6%) and third (14.8%) ETA courses (Figs. 1 and 2). Onset of adverse events was stated as the cause of the discontinuation for 107 (6.2%), 19 (5.9%), and 3 (5.6%) patients for the three ETA courses. Infections (17.2%), eye disorders (15.5%), and gastrointestinal disorders (10.3%) were the most common adverse events that led to ETA discontinuation in the first course. Discontinuation of ETA due to ineffectiveness was not significantly associated with any JIA disease characteristics, such as JIA category or ANA positivity. About 80% of the patients were treated with ETA in the previous therapy course before ETA was started in the second (*n* = 283, 83.7%) and third treatment courses (*n* = 43, 79.6%),, respectively (Fig. 2). The majority of patients switched to ADA after ETA discontinuation due to ineffectiveness, adverse events, and other reasons (1st: *n* = 308, 43.4%; 2nd: *n* = 59, 42.8%), followed by TOC (1st: *n* = 40, 5.6%; 2nd: *n* = 15, 10.9%).

### Response to therapy

**Fig. 3:**
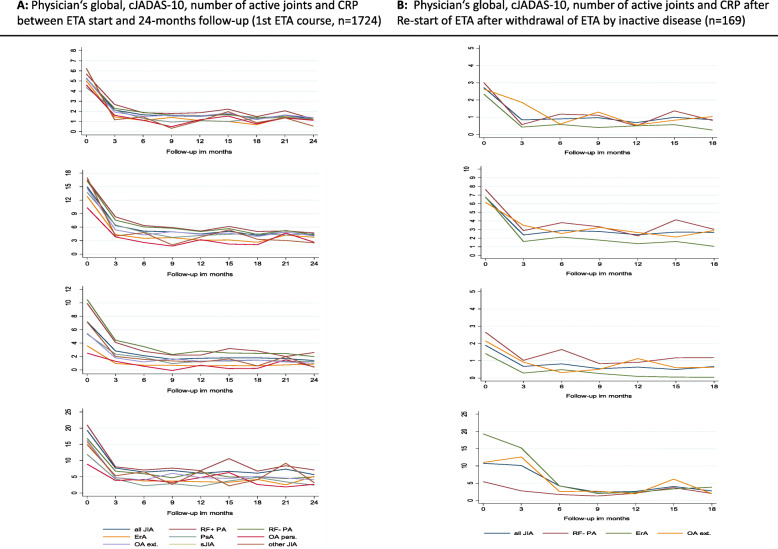
Effectiveness of etanercept (cJADAS-10, number of active joints and CRP), **a** After initiation of treatment in the first ETA course and **b** after etanercept discontinuation due to inactive disease and start of re-treatment

A total of 446 patients (61.5%) were in a state of an inactive disease at the 12-month follow-up, with the highest rates reported for enthesitis-related arthritis (*n* = 95, 68.8%) and persistent oligoarthritis (*n* = 18, 66.7%), and the lowest rate for systemic JIA (*n* = 18, 47.4%) during the first ETA course. The cJADAS-10 score, number of joints with active arthritis, and the presence of CRP in the 24-month follow-up after the start of the first ETA are shown in Fig. 3a.

Three hundred and thirty-two patients (19.3%) discontinued ETA after achieving an inactive disease during the first ETA course in mean after 2.5 (SD 1.6) years. Among those, 222 patients (66.9%) discontinued ETA monotherapy, and the other 110 patients (33.1%) stopped ETA before stopping MTX treatment. A higher likelihood for discontinuing ETA due to achieving an inactive disease was significantly associated with a shorter duration between JIA onset and the start of ETA treatment (hazard ratio (HR) = 0.91, 95% CI 0.87–0.95), lower age at the start of ETA (HR = 0.92, 95% CI 0.89–0.96), higher response to therapy within the first 6 months (HR = 1.12, 95% CI 1.07–1.16), and persistent oligoarthritis (HR = 1.89, 95% CI 1.22–2.93), whereas patients with RF-positive polyarthritis (HR = 0.56, 95% CI 0.35–0.89) were less likely to discontinue ETA due to the onset of inactive disease as per the multivariable analyses (Table 3). The results of the univariable analyses are reported in Supplemental Table 1.

**Table 3:** Correlates of etanercept withdrawal after achieving inactive disease (multivariable model)

	Eta was not withdrawn by inactive disease, *n* = 1392	Eta was withdrawn by inactive disease, *n* = 332	HR	p value	95% CI
Female gender	945 (67.9%)	201 (60.5%)	0.96	0.749	0.73 to 1.25
Age, years, mean (SD)	13.7 (3.5)	11.9 (3.1)	**0.92**	**< 0.001**	**0.89 to 0.96**
ANA positive	589 (42.5%)	128 (38.7%)	0.82	0.148	0.62 to 1.07
HLA-B27 positive	368 (26.6%)	104 (31.4%)	1.10	0.57	0.78 to 1.55
JIA categories					
Systemic JIA	96 (6.9%)	15 (4.5%)	0.83	0.512	0.47 to 1.45
Polyarticular arthritis, RF-negative	386 (27.7%)	101 (30.4%)	1.08	0.572	0.83 to 1.39
Polyarticular arthritis RF-positive	156 (11.2%)	20 (6.0%)	**0.56**	**0.014**	**0.35 to 0.89**
Persistent Oligoarthritis	48 (3.5%)	25 (7.5%)	**1.89**	**0.004**	**1.22 to 2.93**
Extended Oligoarthritis	247 (17.7%)	60 (18.1%)	1.08	0.648	0.79 to 1.47
Enthesitis-related arthritis	286 (20.6%)	73 (22.0%)	1.24	0.197	0.89 to 1.71
Psoriatic arthritis	126 (9.1%)	22 (6.6%)	0.89	0.582	0.59 to 1.35
Duration between JIA onset and bDMARD start, in months, mean (SD)	60.8 (48.5)	46.0 (38.3)	**0.91**	**< 0.001**	**0.87 to 0.95**
c-JADAS-10					
At therapy start	15.1 (6.7)	14.5 (6.5)	**0.9**	**< 0.001**	**0.86 to 0.93**
Therapy response within the first 6 months	9.3 (6.6)	11.3 (6.6)	**1.12**	**< 0.001**	**1.07 to 1.16**

*ANA* antinuclear antibodies, *bDMARD* biological disease-modifying anti-rheumatic drug, *CI* confidence interval, *cJADAS* clinical Juvenile Arthritis Disease Activity Score, *HLA* human leukocyte antigen, *HR* hazard ration, *RF* rheumatoid factor, *SD* standard deviation

### Reoccurrence of active disease after ETA discontinuation

The clinical course of 209 patients (94.1%; *n* = 222) with available follow-up and who discontinued ETA monotherapy after achieving an inactive disease could be investigated over an average time of 3.9 (SD 3.5) years (Fig. 1, lower panel). A total of 161 patients (77.0%) experienced a reoccurrence of an active disease and/or required restarting of a DMARD therapy (*n* = 136, 84.5%). The likelihood for reoccurrence of active disease was not significantly associated with the time in inactive disease before ETA discontinuation (Fig. 1) and other clinical parameters such as JIA category, gender, ANA, and HLA-B27 positivity, response to therapy to ETA within the first 6 months, duration between JIA onset and start of DMARD treatment and the cumulative time under MTX or ETA before ETA discontinuation after achieving inactive disease. The majority of patients with a reoccurrence of active disease were re-treated with ETA (*n* = 117 of 161; 72.7%). Among those, one in five patients (*n* = 23, 19.7%) discontinued ETA again after achieving an inactive disease during the mean follow-up of 2.8 (SD 2.5) years. Approximately 70% of the patients who were re-treated with ETA achieved an inactive disease 12 months after restarting ETA (Fig. 1, lower panel). On average, the patients responded well to reinitiation of ETA treatment, as evidenced by the cJADAS-10 score, number of joints with active arthritis, and existence of CRP in the 18-month follow-up after restarting ETA (Fig. 3b).

## Discussion

Biologic DMARDs have become an integral part of the treatment of patients with moderate to severe JIA. ETA was the first biologic approved for treatment of polyarticular JIA. Despite the growing availability of other bDMARDs during the last 20 years, ETA is still the most commonly prescribed bDMARD [5, 6] in JIA. Here, we presented data showing the therapy survival of ETA, response to treatment, and effectiveness for the first, second, and third ETA treatment courses in a large cohort of patients with JIA. The strength of the study was the long follow-up from enrolment in BiKeR until the last available follow-up in young adulthood in JuMBO. Because of the large cohort and significant time of follow-up, for the first time, we are able to analyze the effectiveness of a second ETA course after discontinuing ETA due to inactive disease in patients with reoccurrence of active disease in the follow-up. We previously reported on the safety of ETA in children, adolescents, and young adults with JIA in the last few years [5, 12, 20–24] in detail; thus, we did not present data about treatment-emergent adverse events in this report.

Interestingly, the likelihood of discontinuing ETA due to an inactive disease was positively associated with a shorter duration between JIA onset and start of ETA among other factors in the multivariable analysis. This result supports the concept of a window of opportunity in JIA, in that early initiation of treatment allows modulation of biologic processes, resulting in more favorable long-term disease trajectories [25, 26]. Recently, we used data from BiKeR and JuMBO to show that early initiation of treatment with a bDMARD was associated with better disease control and outcomes in young adulthood [27], such as a higher rate of patients in drug-free remission or inactive disease, and fewer functional limitations, arthroplasties, and eye surgeries. Initially, the rationale of the window-of-opportunity concept was investigated in patients with rheumatoid arthritis [28, 29], and its existence is widely accepted in the rheumatology community [25, 26]. Closely related to the concept of a window of opportunity is the so-called treat-to-target strategy. It includes an early and aggressive therapy, and if necessary, escalation and adjustment of treatments to reach and maintain specific treatment goals is possible, thus resulting in a tight disease control [3, 30–32]. This concept is indirectly also supported by our study. Patients who responded well to therapy within the first 6 months of treatment were more likely to discontinue ETA after achieving an inactive disease. However, we did not further investigate the clinical course of patients who did not respond to ETA, or discontinued ETA and switched to another bDMARD, as doing so was beyond the scope of this study.

Our data confirm the known effectiveness of ETA in the treatment of JIA. To our knowledge, this is the first prospective study in JIA patients that showed the effectiveness of ETA in patients who required re-treatment with ETA after its discontinuation after achieving an inactive disease. Physicians may hesitate to discontinue ETA after achieving inactive disease because of the flare risk, and if this occurs, the uncertainty about the effectiveness of ETA in re-treatment. ETA seems to be equally effective in re-treatment; every fifth patient could discontinue ETA again due to achieving an inactive disease, and approximately 70% achieved an inactive disease 12 months after restarting ETA. Although with a very limited number of patients, Postepski et al. [33] already reported about a satisfactory treatment response of 12 patients who re-started ETA after JIA flare in a retrospective chart review in 2 centers in Poland.

About 20% of the patients could discontinue ETA after achieving an inactive disease state. We only analyzed the group of patients who discontinued ETA monotherpay, i.e., MTX was first discontinued in patients with combination of ETA and MTX at treatment start. The primary aim of our study was to show the effectiveness of ETA even in the re-treatment with ETA, rather than comparing the discontinuation strategies MTX or ETA first in patients with combination therapy. This is a more general question, concerns all bDMARDs, and is not limited only to ETA. The analysis of the two discontinuation strategies MTX or bDMARDs first is methodologically challenging in cohort studies, because of the imbalance of patient characteristics between the two groups resulting in the bias by indication. Further adjustments in statistics by propensity scores [34] or the inclusion of time-varying covariates [35] are necessary in order to get a fair comparison in flare rates between the two groups.

Our analysis confirmed a high flare rate of JIA as observed by others [36–41]. A total of 161 patients (77%) experienced reoccurrence of active disease or were required to restart DMARD treatment in a follow-up when discontinuing ETA monotherapy and MTX first in combination therapy with ETA, respectively. Chang et al. [36] reported similar flare rates (76%) within 24 months after entering inactive disease and subsequent discontinuation of a bDMARD. A slightly lower flare rate was reported for MTX monotherapy after discontinuation (56–63%) [36–38]; however, the flare rate after MTX discontinuation is also high. It is consistently reported in literature that flare rates are lower for patients treated with MTX only compared to patients who require a bDMARD to control the disease [36, 39]. However, higher flare rates in patients treated with bDMARDs may partially explained by the more severe JIA disease than patients treated with MTX only. Chang et al. [36] compared in their large single-center retrospective study flare rates for patients who stopped MTX (19%) or bDMARD (78%) first in combination therapy after 12 months. This result was consistently found in the JIA categories RF-positive and RF-negative polyarticular JIA and ERA and they suggested to discontinue MTX first in combination therapy in JIA based on the study data. Two thirds of patients stopped MTX first in combination therapy after achieving inactive disease in our cohort. It may reflect the widely used withdrawal strategy in Germany. Withdrawal of MTX first is also the preferred strategy in the USA and Canada [42].

Several parameters predisposing for disease flare after discontinuation of treatment were identified in retrospective data analyses for a small number of patients in the literature. RF-positive polyarthritis [39], high disease activity during the disease course (as evidenced by high physicians’ global assessments and a high number of affected joints) [39], and ANA positivity [39, 43] were associated with a higher flare risk. A recent prospective study of Lovell et al. [40] identified a shorter disease duration at study enrolment and shorter time from disease onset until first occurrence of inactive disease as well as later age at JIA onset as predictors for a decreased flare risk. In contrast to all previous study results, we could not find any significant predicting variable for the flare risk. Some references to predictors of flares could be found in the literature, but all the studies [39, 40, 43] identified different parameters, and the conditions of these studies were heterogeneous.

Pediatric rheumatologists continue to debate over whether the time in inactive disease before bDMARD discontinuation predicts the flare risk. Simonini et al. [43] were the first to analyze retrospective chart review data of patients who continue treatment for more than 2 years after achieving an inactive disease before discontinuing treatment. These patients had a significantly lower risk for reoccurrence of active disease. It should be noted that the number of patients followed-up was particularly low. With a very much higher number of patients, we could not confirm this finding in our analyses. A time of more than 2 years in inactive disease before discontinuation of ETA may be slightly favorable within the first 24 months after discontinuation (Fig. 1), but the flare rates become similar with further follow-ups. Lovell et al. [40] even showed a higher likelihood for disease flares in patients with longer durations in inactive disease.

Only half of the patients who initiated treatment with ETA in combination with MTX were still on combination therapy for all three ETA cycles at ETA discontinuation or last follow-up. The proportion of patients (70 to 28%) and the mean time under combination therapy (1.5 to 0.2 years) decreased remarkably from first to third ETA cycle. The reasons for discontinuation of concomitant treatment with MTX were not documented for most patients. We may speculate that MTX intolerance could be one of the reasons for discontinuation of MTX because of unwanted gastrointestinal adverse events in a larger proportion of patients in our study [44–46]. It is estimated that up to 50% of patients with JIA develop a MTX intolerance [47]. MTX intolerance often occurs within the first year of treatment and get worse [46]. Van der Meer et al. [46] reported a proportion of patients with MTX intolerance of about 25% and 30% after 6 and 12 months of MTX treatment. MTX intolerance may be also a reason that two third of patients discontinued MTX first in combination therapy after achieving an inactive disease.

This study also suffers from the following limitations. BiKeR and JuMBO are observational studies reflecting the real-life treatments of patients with JIA in specialized care. Our results are not transferable to all patients with JIA. We analyzed patients with moderate to severe JIA who were qualified to receive treatment with a bDMARD. In addition, missing values and lost-to-follow-up patients may introduce a small selection bias towards patients with a more severe JIA course. Our analysis focused on the discontinuation of ETA after achieving inactive disease. The individual decision for the discontinuation of ETA was made by the pediatric or adult rheumatologist rather than by chance in the observational study design. However, the impact of a missing protocol to discontinue treatment may be minimized by the large variation in treatment decisions by the high number of participating rheumatology centers. Our study also benefited from certain strengths, namely the prospective study design of a tightly monitored cohort, the inclusion of a large number of patients, and a long follow-up into adulthood.

The main focus of our study was on the effectiveness of ETA. Consequently, only the discontinuation of ETA was analyzed in patients with ETA monotherapy or patients who discontinued MTX before ETA in combination therapy in order to show the effectiveness of ETA re-treatment. To date, only one large study (CHANG) compared the outcome of different medication withdrawal strategies including MTX and bDMARD in monotherpay and MTX or bDMARD first in combination therapy. Future studies should focus on comparing medication withdrawal strategies including the tapering of medications in large cohorts.

## Conclusion

In conclusion, the results of this study confirmed the effectiveness of ETA, even in the re-treatment of patients with JIA who discontinued ETA after achieving an inactive disease. The discontinuation of ETA due to inactive disease was positively associated with a shorter time between JIA onset and start of treatment with ETA and a good response to therapy within the first 6 months. Thus, the presence of a window of opportunity may be further supported by our study.

## Supplementary Information

**Additional file 1: Supplemental Table 1**. Univariable correlates of etanercept withdrawal after achieving inactive disease.

## Data Availability

None
