# Supplementary material for: Re-treatment with etanercept is as effective as the initial firstline treatment in patients with juvenile idiopathic arthritis
Source: Arthritis Res Ther. 2021 Apr 16;23:118. doi: 10.1186/s13075-021-02492-0 (PMC8050932; doi:10.1186/s13075-021-02492-0)
Supplement: Supplementary file 1 — Additional file 1: Supplemental Table 1. Univariable correlates of etanercept withdrawal after achieving inactive disease. [file 13075_2021_2492_MOESM1_ESM.docx]

Supplemental table1: Univariable correlates of etanercept withdrawal after achieving inactive disease

|  |  |  |  |  |  |  |  |
| --- | --- | --- | --- | --- | --- | --- | --- |
|  |  | Eta was not withdrawn by inactive disease | Eta was withdrawn by inactive disease | HR | p value | 95%CI | c statistics |
|  |  | n=1392 | n=332 |  |  |  |  |
|  |  |  |  |  |  |  |  |
| Female gender | | 945 (67.9%) | 201 (60.5%) | 0.78 | 0.028 | 0.63 to 0.97 | 0.53 |
| Age, years, mean (SD) | | 13.7 (3.5) | 11.9 (3.1) | 0.92 | <0.001 | 0.89 to 0.95 | 0.58 |
|  |  |  |  |  |  |  |  |
| JIA categories | |  |  |  |  |  |  |
|  | Systemic JIA | 96 (6.9%) | 15 (4.5%) | 0.75 | 0.227 | 0.48 to 1.19 | 0.58 |
|  | Polyarticular arthritis, RF-negative | 386 (27.7%) | 101 (30.4%) | 1.01 | 0.922 | 0.81 to 1.26 |  |
|  | Polyarticular arthritis RF-positive | 156 (11.2%) | 20 (6.0%) | 0.52 | 0.002 | 0.34 to 0.78 |  |
|  | Persistent Oligoarthritis | 48 (3.5%) | 25 (7.5%) | 2.19 | <0.001 | 1.52 to 3.15 |  |
|  | Extended Oligoarthritis | 247 (17.7%) | 60 (18.1%) | 0.97 | 0.839 | 0.75 to 1.26 |  |
|  | Enthesitis-related arthritis | 286 (20.6%) | 73 (22.0%) | 1.22 | 0.1 | 0.96 to 1.56 |  |
|  | Psoriatic arthritis | 126 (9.1%) | 22 (6.6%) | 0.83 | 0.338 | 0.56 to 1.22 |  |
|  |  |  |  |  |  |  |  |
| Duration between JIA onset and bDMARD start, in months, mean (SD) | | 60.8 (48.5) | 46.0 (38.3) | 0.9 | <0.001 | 0.87 to 0.93 | 0.6 |
|  |  |  |  |  |  |  |  |
| ANA positive | | 589 (42.5%) | 128 (38.7%) | 0.82 | 0.091 | 0.66 to 1.03 | 0.53 |
| HLA-B27 positive | | 368 (26.6%) | 104 (31.4%) | 1.37 | 0.01 | 1.08 to 1.74 | 0.56 |
|  |  |  |  |  |  |  |  |
| Physician's global assessment, VAS score, mean (SD) | |  |  |  |  |  |  |
|  | At therapy start | 5.4 (2.7) | 5.1 (2.6) | 0.71 | <0.001 | 0.65 to 0.79 | 0.65 |
|  | Therapy response within the first 6 months | 3.4 (2.7) | 4.1 (2.6) | 1.39 | <0.001 | 1.26 to 1.53 |  |
| c-JADAS-10, mean (SD) | |  |  |  |  |  |  |
|  | At therapy start | 15.1 (6.7) | 14.5 (6.5) | 0.88 | <0.001 | 0.85 to 0.91 | 0.63 |
|  | Therapy response within the first 6 months | 9.3 (6.6) | 11.3 (6.6) | 1.14 | <0.001 | 1.10 to 1.18 |  |
| Number of joints with arthritis, mean (SD) | |  |  |  |  |  |  |
|  | At therapy start | 7.2 (8.6) | 7.1 (8.8) | 0.87 | <0.001 | 0.82 to 0.92 | 0.57 |
|  | Therapy response within the first 6 months | 4.7 (7.3) | 6.0 (7.8) | 1.16 | <0.001 | 1.09 to 1.24 |  |
| Number of LOM joints, mean (SD) | |  |  |  |  |  |  |
|  | At therapy start | 8.2 (9.8) | 7.2 (9.5) | 0.91 | <0.001 | 0.89 to 0.94 | 0.61 |
|  | Therapy response within the first 6 months | 3.7 (7.2) | 4.8 (8.2) | 1.11 | <0.001 | 1.07 to 1.15 |  |
| Number of painful joints, mean (SD) | |  |  |  |  |  |  |
|  | At therapy start | 7.3 (9.0) | 6.7 (9.1) | 0.88 | <0.001 | 0.83 to 0.93 | 0.58 |
|  | Therapy response within the first 6 months | 4.7 (7.9) | 5.5 (8.6) | 1.15 | <0.001 | 1.08 to 1.21 |  |
| Number of swollen joints, mean (SD) | |  |  |  |  |  |  |
|  | At therapy start | 5.6 (7.8) | 5.8 (8.2) | 0.88 | <0.001 | 0.83 to 0.94 | 0.56 |
|  | Therapy response within the first 6 months | 3.8 (6.6) | 4.9 (7.4) | 1.15 | <0.001 | 1.07 to 1.22 |  |
| CHAQ total score, mean (SD) | |  |  |  |  |  |  |
|  | At therapy start | 0.71 (0.63) | 0.61 (0.60) | 0.34 | <0.001 | 0.23 to 0.50 | 0.61 |
|  | Therapy response within the first 6 months | 0.33 (0.45) | 0.40 (0.51) | 3.57 | <0.001 | 2.29 to 5.59 |  |
| Patient-reported overall well-being, VAS, mean (SD) | |  |  |  |  |  |  |
|  | At therapy start | 4.7 (2.7) | 4.5 (2.8) | 0.83 | <0.001 | 0.76 to 0.90 | 0.6 |
|  | Therapy response within the first 6 months | 2.7 (2.7) | 3.3 (2.9) | 1.21 | <0.001 | 1.11 to 1.32 |  |
| Patient-reported pain, VAS, mean (SD) | |  |  |  |  |  |  |
|  | At therapy start | 4.4 (2.6) | 3.8 (2.6) | 0.79 | <0.001 | 0.73 to 0.86 | 0.61 |
|  | Therapy response within the first 6 months | 2.4 (2.5) | 2.6 (2.6) | 1.24 | <0.001 | 1.13 to 1.36 |  |
| ANA = antinuclear antibodies; bDMARD = biological disease-modifying antirheumatic drug; CI = confidence interval; CHAQ = Childhood Health Assessment Questionnaire; cJADAS = clinical Juvenile Arthritis Disease Activity Score; CRP = C reactive protein; ESR = erythrocyte sedimentation rate; HLA = human leukocyte antigen; HR = hazard ratio; LOM = limitation of motion; RF = rheumatoid factor; SD = standard deviation; VAS = visual analogue scale | | | | | | | |
